# An Uncommon Presentation of a Solid Pseudopapillary Neoplasm in a Male Patient: Diagnostic Challenges and Multidisciplinary Management of a Pancreatic Tail Mass

**DOI:** 10.7759/cureus.107270

**Published:** 2026-04-17

**Authors:** Rana Shalaby, Khadija Jaffar KI Radwani, Sarah Sayed, Mohamed Lameir Mukhtar Hussein

**Affiliations:** 1 Radiology, Hamad Medical Corporation, Doha, QAT; 2 Pathology, Hamad Medical Corporation, Doha, QAT

**Keywords:** cystic pancreatic tumour, epithelial tumor, pancreatic exocrine neoplasms, pancreatic tail tumor, rare pancreatic tumor, solid pseudopapillary neoplasm

## Abstract

Solid pseudopapillary neoplasm (SPN) is a rare pancreatic tumor that typically affects young women and has a low malignant potential. We report the case of a 37-year-old man who was being investigated for secondary hypertension when imaging revealed a pancreatic tail mass. Radiological findings suggested a differential diagnosis including SPN, cystic adenocarcinoma, or a mesenchymal tumor. Further evaluation with MRI, PET, and histopathology confirmed the diagnosis of SPN. The patient underwent successful robotic distal pancreatectomy with splenectomy and was found to have an R0 resection with no nodal involvement. This case highlights the importance of a multidisciplinary approach and maintaining a broad differential diagnosis, even in atypical patient populations.

## Introduction

Solid pseudopapillary neoplasm (SPN) of the pancreas is a rare epithelial tumor that accounts for less than 2% of all pancreatic exocrine neoplasms [1]. First described by Frantz in 1959, SPNs have a predilection for young women in their second to third decades of life, with a female-to-male ratio of approximately 10:1 [1]. Although the majority of SPNs are asymptomatic and diagnosed incidentally via different imaging modalities such as computed tomography (CT) or magnetic resonance imaging (MRI), patients may present with non-specific abdominal pain or discomfort [2]. These tumors typically have low malignant potential, and complete surgical resection offers an excellent prognosis [3]. However, when SPNs present in atypical demographics such as males or older patients, diagnosis can be delayed or overlooked. This case report presents an uncommon presentation of SPN in a middle-aged man, which was incidentally discovered during evaluation for secondary hypertension.

## Case presentation

A 37-year-old man with a medical history of hypertension, hyperlipidemia, and asthma was diagnosed with hypertension in 2022 in his home country. In 2023, he was referred to an Internal Medicine outpatient clinic for evaluation of secondary hypertension. Since diagnosis, he remained asymptomatic, without complaints of headache, dizziness, or visual disturbances. His vital signs and physical examination were unremarkable, with no palpable masses or signs of organomegaly.

**Figure 1:**
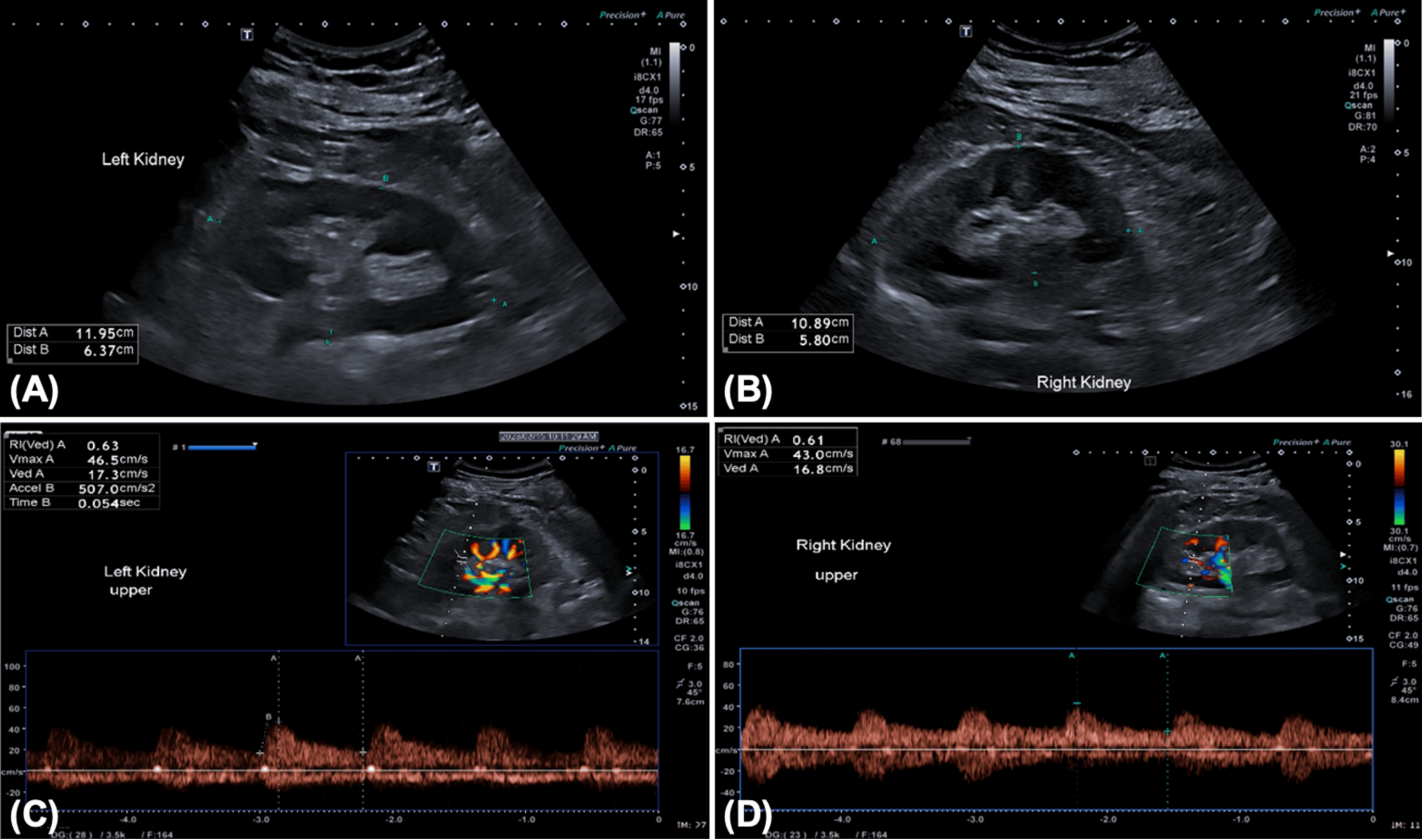
Grey-scale (A and B) and colour Doppler (C and D) ultrasound images showing bilateral normal-sized kidneys with normal echogenicity, cortical thickness, and duplex parameters.

Laboratory investigations revealed elevated plasma aldosterone (670 pmol/L), suppressed plasma renin activity (0.25 ng/mL/hr), and an elevated aldosterone-to-renin ratio (96), suggesting primary hyperaldosteronism (as shown in Table 1). Renal Doppler ultrasound showed no evidence of renal artery stenosis (Figure 1). The patient was referred to the endocrinology clinic, and an abdominal CT was requested to evaluate for adrenal pathology.

**Table 1:** Laboratory investigations suggesting primary hyperaldosteronism.

Laboratory test	Result	Reference range
Plasma aldosterone	670 pmol/L	48.9-644.4 pmol/L
Plasma renin activity	0.25 ng/mL/hr	0.29-6.12 ng/mL/hr
Aldosterone-to-renin ratio	96	Aldosterone-to-renin ratio ≥ 20 and plasma aldosterone ≥ 417 pmol/L indicate probable primary aldosteronism

**Figure 2:**
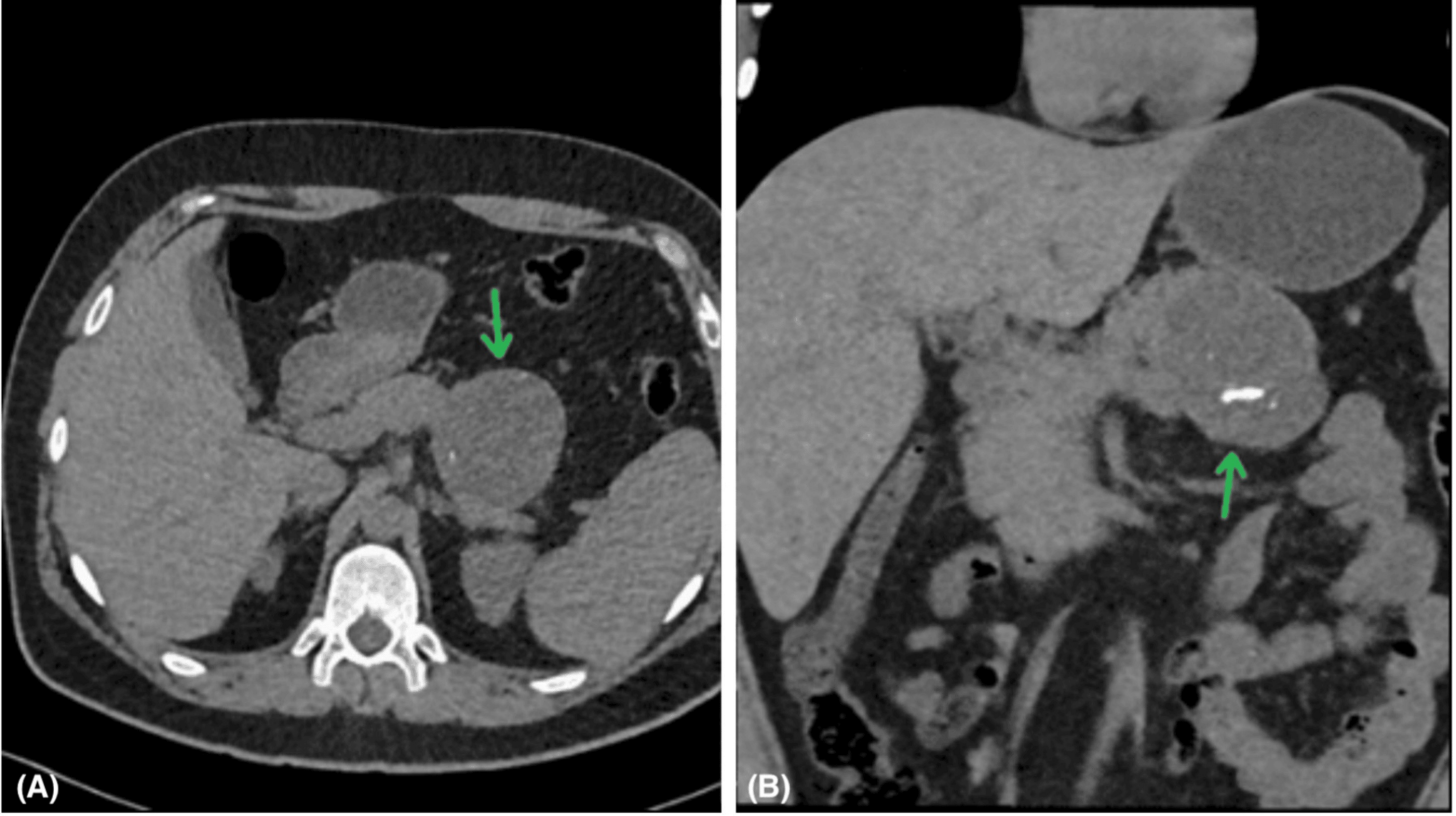
Axial (A) and coronal (B) plane CT scan showing a well-defined low attenuation mass centered in the pancreatic tail region with intralesional speckles of calcification (green arrows).

CT imaging revealed a 6.4 x 5.7 cm well-defined, rounded, mildly hypodense lesion with internal calcifications and an attenuation of 19-22 HU (Figure 2). The lesion was closely related to the body and tail of the pancreas and abutted the gastric wall, raising a suspicion of pancreatic origin. However, an exophytic adrenal lesion could not be excluded.

**Figure 3:**
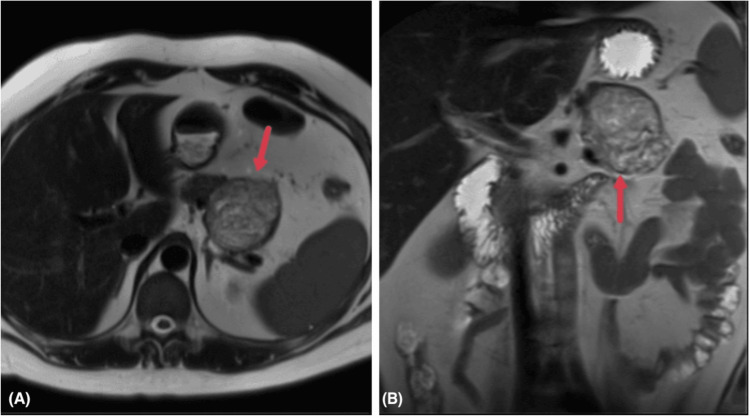
MRI of the abdomen: axial T2W (A) and coronal T2W (B) images showing the well-defined mass arising from the pancreatic tail with a heterogeneous intermediate-to-high T2 signal with a hypointense capsule (red arrows).

**Figure 4:**
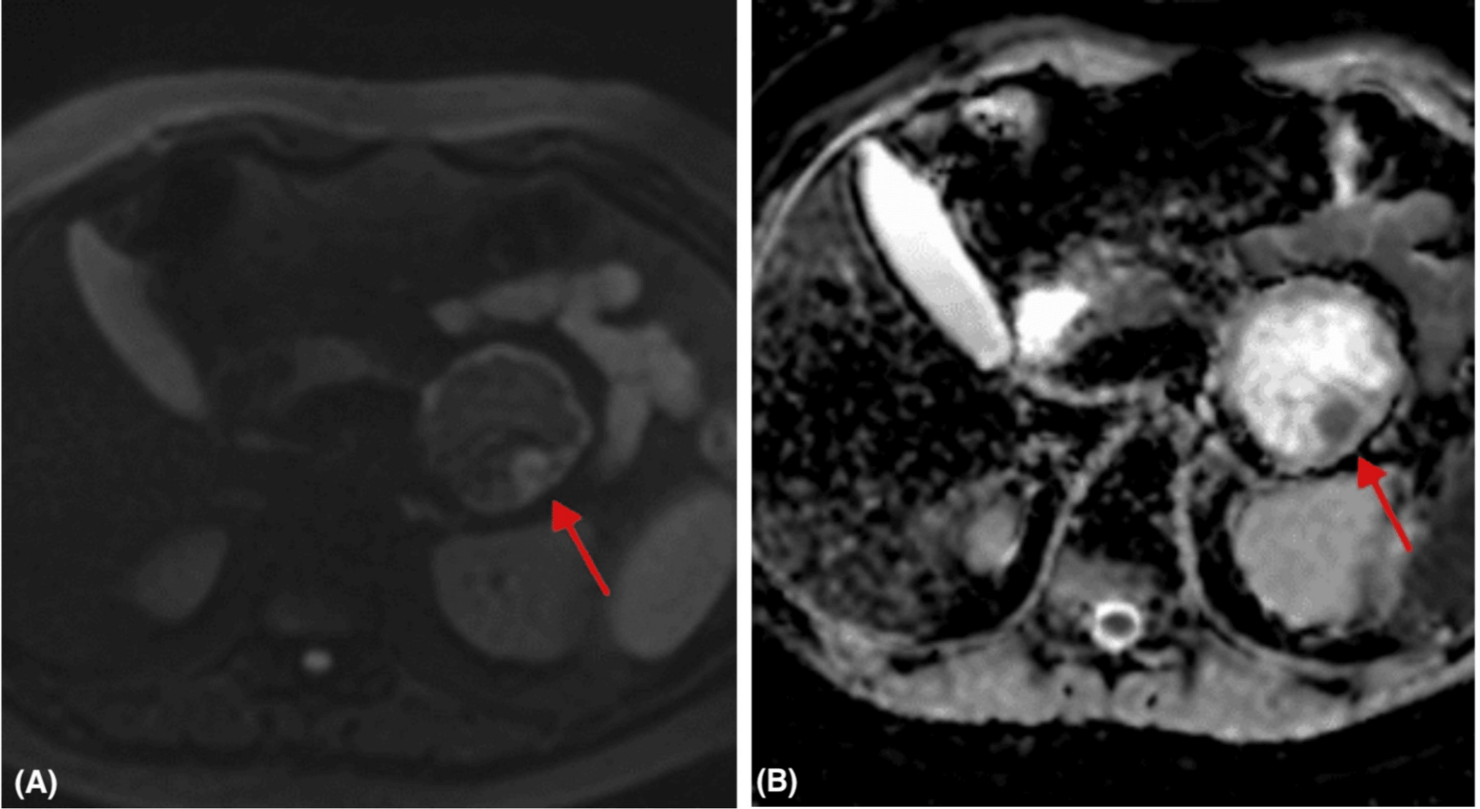
MRI of the abdomen: axial high-B value diffusion (A) and ADC (B) images showing wall and nodular diffusion restriction (red arrows). ADC: apparent diffusion coefficient

**Figure 5:**
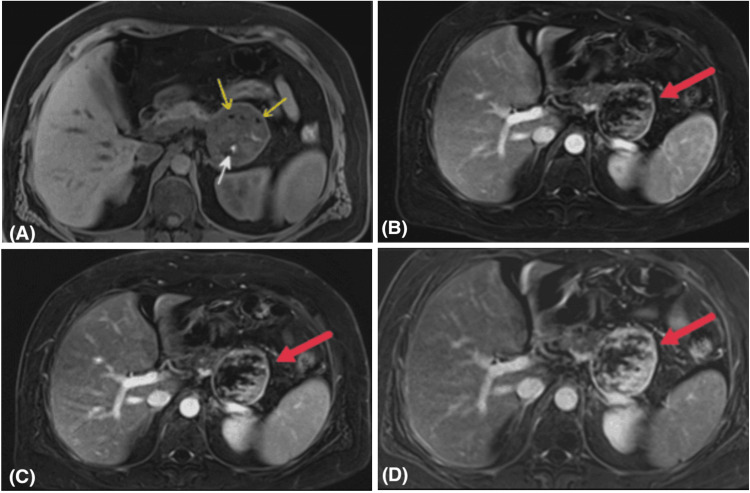
MRI of the abdomen: axial plane T1 fat saturation (sat) (A), arterial phase T1 fat sat (B), venous phase T1 fat sat (C), and delayed phase T1 fat sat (D) images showing the well-defined mass arising from the pancreatic tail with fat foci (yellow arrows), hemorrhagic foci (white arrow) and progressive heterogeneous enhancement (red arrows).

MRI was subsequently performed and demonstrated a well-circumscribed, solid-cystic mass in the pancreatic tail measuring 62.4 x 61.3 x 61.5 mm, showing heterogeneous high T2 signal (Figure 3), peripheral and nodular high signal on high-B value diffusion sequence and low signal on apparent diffusion coefficient (ADC) sequences (Figure 4), low T1 signal with intralesional hemorrhagic foci and fat foci, and post-contrast progressive heterogeneous enhancement (Figure 5). The mass was in close contact with the splenic vessels, although they remained patent. No peripancreatic fluid collection or pancreatic duct dilatation was noted. Radiological differential diagnoses included SPN, adenocarcinoma with cystic degeneration, and mesenchymal tumors.

**Figure 6:**
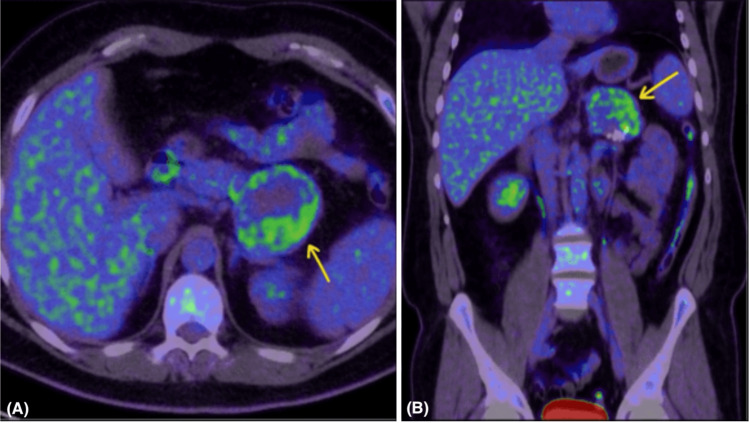
FDG PET/CT images: axial (A) and coronal (B) views of CT soft tissue window showing the pancreatic tail hypodense mass with peripheral and nodular uptake more at the posterior and lateral aspect (yellow arrows). PET/CT: positron emission tomography/computed tomography

The case was reviewed in the hepatobiliary multidisciplinary team (MDT) meeting, which recommended positron emission tomography (PET) and surgical intervention. PET/CT showed mild-to-moderate FDG uptake, predominantly peripheral, without definitive evidence of metastasis (Figure 6).

**Figure 7:**
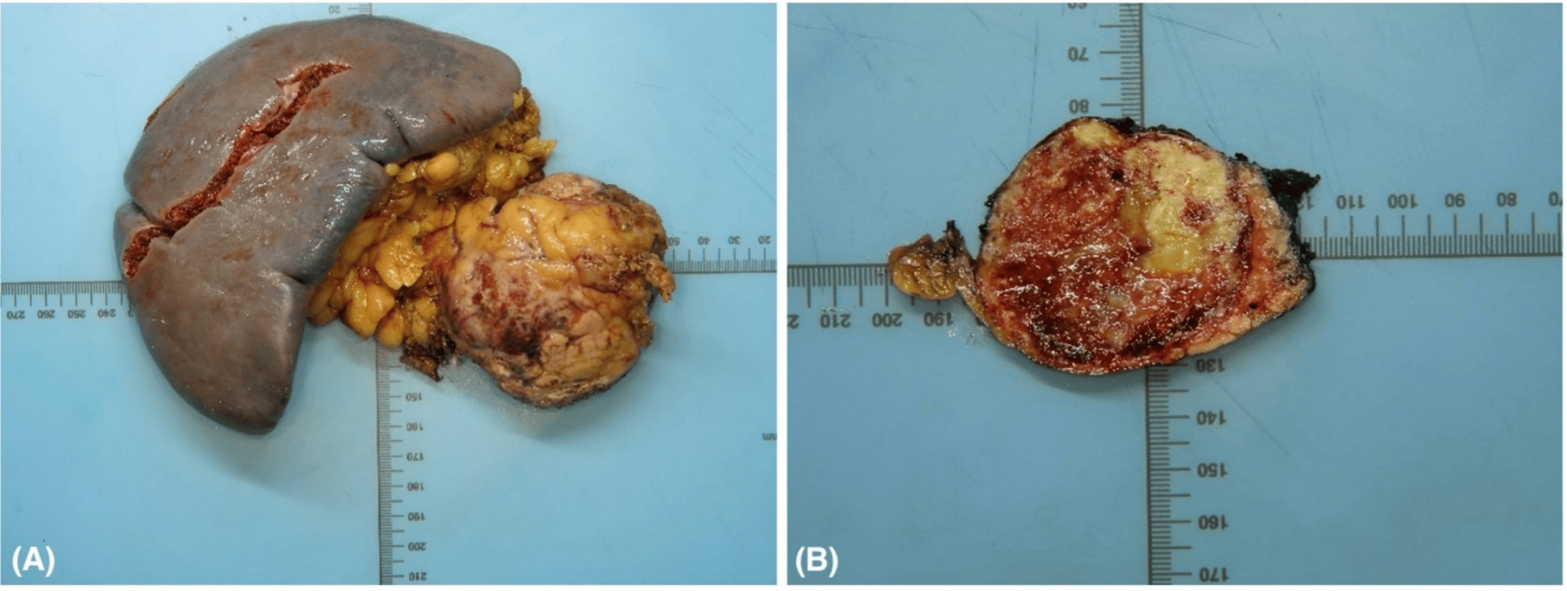
Gross image of the resected pancreatic tail mass, the splenic hilar soft tissue, and the resected spleen (A). The mass does not involve the spleen grossly but appears to be abutting the splenic hilar tissue. Cross sections of the pancreatic tail mass (B) show a heterogeneous surface with mixed bright yellow areas and hemorrhagic cystic areas reflecting the different morphologies microscopically.

The patient underwent robotic distal pancreatectomy with splenectomy. On gross examination, the specimen weighed 599 g. Serial sectioning of the pancreas revealed a well-circumscribed lesion located in the distal portion, measuring 6.5 × 5.5 × 5.5 cm. The cut surface of the mass was heterogeneous, displaying tan-yellow areas, foci of hemorrhage, light brown fleshy zones, and white calcified regions, reflecting the mixed gross and microscopic appearance characteristic of this tumor (Figure 7). The lesion was found abutting the splenic capsule, which appeared focally disrupted.

**Figure 8:**
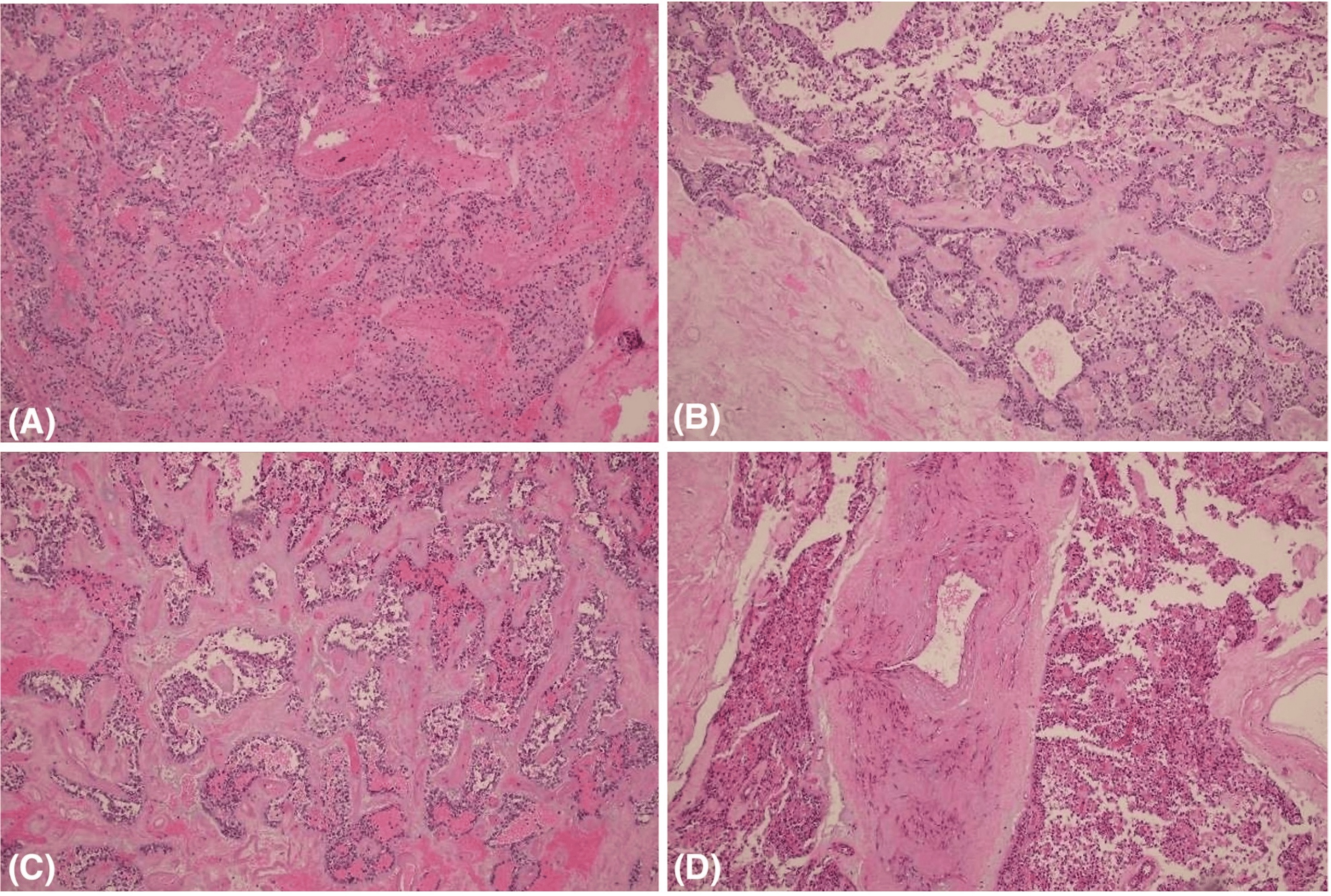
Histopathological features of the pancreatic solid pseudopapillary tumor: solid areas with monotonous cells with a hyalinized degenerative background (A); pseudopapillary areas with a myxoid hyalinized background (B-C); infiltration of tumor into the hilar splenic tissue (D).

Microscopically, the tumor showed heterogeneous architecture. It consisted of monotonous solid sheets of cells arranged around blood vessels, admixed with pseudo-papillary structures where the cells detached from the vascular core (Figure 8). Cytologically, the tumor cells displayed uniform nuclei, fine chromatin, inconspicuous nucleoli, and characteristic nuclear grooves. Mitotic figures were rare. The background demonstrated marked hyalinization and areas of hemorrhage, indicating degenerative changes (Figure 8).

These histomorphological features are diagnostic of a solid pseudopapillary neoplasm. Although immunohistochemical staining (IHC) can aid in the diagnosis, typically showing strong nuclear β-catenin, SOX11, CD10, vimentin, cyclin D1, and pancreatic markers such as alpha-1-antichymotrypsin and alpha-1-antitrypsin, IHC was not performed in this case, as the diagnosis was confidently made based on the characteristic histological features.

Importantly, the tumor did not exhibit high-risk features suggestive of aggressive behavior, such as high mitotic activity, extensive necrosis, marked nuclear atypia, or sarcomatoid transformation. There was evidence of infiltration into vascular tissue near the splenic hilum, but no direct invasion of the spleen was identified. The pancreatic resection margin was clear, with a 5 mm distance from the tumor.

The final histopathological evaluation confirmed a solid pseudopapillary neoplasm with R0 resection margins and no lymph node involvement (pT3pN0). The patient was discharged without complications and was scheduled for surveillance follow-up.

## Discussion

SPNs are rare pancreatic tumors with unique clinical, radiological, and pathological features. Despite their rarity, they should be considered in the differential diagnosis of cystic pancreatic lesions, particularly in younger individuals. The majority of SPNs occur in women, with only about 10% of cases reported in males [1]. This demographic distinction may contribute to diagnostic challenges when SPNs present in male patients, as in the current case.

In most patients, SPNs are either asymptomatic or present with non-specific abdominal symptoms such as pain or discomfort [3]. In this case, the pancreatic lesion was discovered incidentally during workup for secondary hypertension. The increasing use of imaging in clinical practice has led to more frequent identification of incidental pancreatic masses, often referred to as “incidentalomas” [4].

Radiologically, SPNs commonly appear as large, well-encapsulated lesions with mixed solid and cystic components, hemorrhagic degeneration, and occasionally calcifications [5]. These features overlap with those seen in other pancreatic lesions such as mucinous cystic neoplasms or pancreatic ductal adenocarcinomas with cystic degeneration [6]. Lack of pancreatic duct dilation and well-defined margins favored a less aggressive neoplasm in this case.

Although PET/CT may demonstrate low-to-moderate FDG uptake, it is not specific for SPNs and cannot reliably distinguish them from malignant pancreatic neoplasms [7]. Thus, surgical excision is recommended for both diagnostic confirmation and curative intent.

Histologically, SPNs exhibit characteristic pseudopapillary architecture, uniform cells with eosinophilic cytoplasm, and frequent areas of hemorrhage and necrosis. Immunohistochemically, they often stain positive for beta-catenin, CD10, vimentin, and progesterone receptors [1]. The prognosis following complete surgical excision is excellent, with five-year survival rates exceeding 95% [1,3]. However, recurrence or metastasis can occur, particularly in cases with vascular invasion or incomplete resection.

## Conclusions

Solid pseudopapillary neoplasm is a rare, low-grade malignancy of the pancreas that should be considered in the differential diagnosis of cystic pancreatic lesions, even in male patients. This case highlights the significance of incidental findings during unrelated clinical workups and the value of multidisciplinary evaluation. Surgical resection remains the cornerstone of treatment, with favorable long-term outcomes in patients undergoing complete excision.
